# Wastewater Surveillance for Early Warning of Infectious Disease Outbreaks: A Systematic Review of Evidence and Implications for One Health Surveillance

**DOI:** 10.3390/pathogens15070690

**Published:** 2026-06-30

**Authors:** Sucharita Panigrahi, Matrujyoti Pattnaik, Rachita Pradhan, Debaprasad Parai, Shishirendu Ghosal, Anoop Velayudhan, Punit Prasad, Adyasha Panda, Debdutta Bhattacharya, Sanghamitra Pati

**Affiliations:** 1 ICMR-National Institute of Health Research, Bhubaneswar 751023, India; sucharita.panigrahi03@gmail.com; 2 Department of Microbiology & One Health, ICMR-National Institute of Health Research, Bhubaneswar 751023, India; matrujyoti.pattnaik@gmail.com (M.P.); rachitapradhan102000@gmail.com (R.P.); debaprasad.bio@gmail.com (D.P.); shishirendu123@gmail.com (S.G.); 3 Academy of Scientific and Innovative Research [AcSIR], Ghaziabad 201002, India; 4 Indian Council of Medical Research, New Delhi 110029, India; anoopvel@gmail.com; 5 DBT-Institute of Life Sciences, Bhubaneswar 751023, India; punit@ils.res.in (P.P.); adyashapanda@ils.res.in (A.P.)

**Keywords:** wastewater surveillance, One Health approach, pathogen surveillance, disease outbreak, infectious diseases

## Abstract

Introduction: Integrated One Health-based surveillance of pathogens in wastewater suggests its potential for monitoring community health and preventing the emergence and spread of infectious diseases. Despite the growing popularity of Wastewater Surveillance (WWS) and its clinical utility, its uniformity remains poorly understood, especially concerning its clinical evidence. This review systematically synthesizes evidence on the role of wastewater surveillance in early pathogen detection and outbreak preparedness, with particular emphasis on its implications for One Health surveillance. Methods: We systematically searched PubMed, EMBASE, ProQuest and EBSCO CINAHL databases. Retrieved articles were screened by two reviewers, and conflicts were resolved by a third reviewer. Initially, 539 studies were retrieved as potentially eligible published articles, of which 16 articles fulfilled the inclusion criteria. Results: Most pathogens identified in the included studies were associated with respiratory and gastrointestinal infections. Studies found a positive link between the presence of pathogens in wastewater and clinical cases, depicting potential exposure and transmission within the communities. A season-specific upsurge was observed among the identified pathogens in circulation. In addition, the duration and frequency of sample collection in socio-vulnerable areas provide early warning of disease outbreaks. Few studies have explicitly operationalized a One Health framework, highlighting the need for integrated human, animal, and environmental surveillance systems in future wastewater surveillance programmes. Conclusion: The review emphasized wastewater surveillance as a promising complementary approach for the early detection and tracking of pathogens. Future research is needed to standardize surveillance approaches and strengthen One Health integration across human, animal and environmental health systems.

## 1. Introduction

Wastewater surveillance (WWS) is a powerful approach to detect the risk of potential pathogens and their genetic material cost-effectively at the population level. It helps to detect and assess spatial and temporal trends of disease-causing pathogens that are generally transmitted by a fecal or waterborne route. In the 1940s, epidemiologists tested sewage water to track and contain polio outbreaks in the United States [1]. WWS was used as a critical tool for eradicating polio worldwide with cell culture methods that are crude by today’s standards but effective. Meanwhile, monitoring the community’s sewage can predict small outbreaks and epidemics of enterovirus and adenovirus disease within the community [2]. In some instances, WWS detected viruses before clinical cases appeared. During the *Coxsackievirus* B5 outbreak, the viruses were identified 10 days prior to the detection of clinical cases. Additionally, Israel’s public health laboratories have been conducting monthly environmental surveillance of sewage since 1989 [3]. This contributed to the recent detection of a wild poliovirus type 1 outbreak in Rahat, Israel in 2013–2014 [4]. Hence, WWS is a potential tool to provide real-time alerts, often days or weeks before any outbreak.

Many of the national and international disease surveillance initiatives focus on a few pathogens in the clinical setting. However, such clinical surveillance requires a large number of patients to obtain meaningful information based on the passive reporting of laboratory testing [5]. Although a clinical-based surveillance approach can measure the prevalence of disease, it is not comprehensive, as it includes only clinical signs and symptoms and hospitalized patients, but does not consider asymptomatic individuals. The data collected from this method can undergo logical constraints and underreporting [6]. These limitations can affect the timely detection of emerging and re-emerging infectious diseases, especially in low-resource settings. Reliance solely on clinical surveillance may not accurately capture the prevalence of disease at the community level. WWS provides a cost-effective and non-invasive way of assessing these deficiencies of traditional surveillance methods [7].

The application of wastewater surveillance extends beyond infectious disease monitoring. It has been utilized to track antimicrobial resistance (AMR), assess community drug use patterns, and monitor environmental pollutants that impact human health [8]. Notably, WWS was integrated with One Health in US, which provided an opportunity to prospectively monitor Influenza from human and animal sources from the same water body [9]. This interconnectedness of human, animal and environmental health under One Health provides a scope for integrating a multidisciplinary approach for enhancing early disease detection and outbreak prevention through wastewater surveillance [10]. Recently, the COVID-19 pandemic has highlighted the necessity of wastewater surveillance through tracking different variants and their prevalence by previously informing about public outbreaks [7]. Through the integration of WWS into the One Health framework, public health authorities can obtain a more comprehensive understanding of disease transmission dynamics and the environmental reservoirs of pathogens [11]. This approach supports timely decision-making, enhances outbreak preparedness, and facilitates targeted interventions to mitigate public health risks [12].

Despite increasing global interest, research on wastewater surveillance remains fragmented due to its study design variations, sampling methodology and interpretation of data. Although various studies have demonstrated the feasibility and efficacy of wastewater surveillance for specific pathogens, there is a need for a systematic synthesis of evidence to assess its overall impact on outbreak prevention and early warning systems. Gaps in standardization, threshold sensitivity and integration of WWS data with traditional public health infrastructure are necessary for the critical evaluation of existing literature.

This review synthesizes evidence on the contribution of wastewater surveillance to infectious disease detection and outbreak preparedness, examining its relevance, opportunities, and challenges within a One Health framework, while identifying gaps in the implementation of formally integrated One Health surveillance systems.

## 2. Materials and Methods

This study was conducted according to the “Preferred Reporting Items for Systematic Reviews and Meta-Analyses” (PRISMA) Guidelines (Supplementary File S1). The study protocol was registered with the international registry “PROSPERO” (registration code CRD42024605101).

### 2.1. Search Strategy

A comprehensive literature search was conducted to identify all electronically published articles, relevant to wastewater pathogen surveillance based on a One Health framework. Databases such as PubMed, Embase, CINHAL, ProQuest were curated using MeSH terms and text related to these three concepts: “Wastewater surveillance”, “Disease outbreak”, and “One health”. Individual search strategies were developed for each database (Supplementary File S2). Also, reference mining was performed to obtain more relevant articles. There was no language restriction in the primary search, but language restriction to English was applied in a further screening process.

### 2.2. Inclusion/Exclusion Criteria

Articles reporting wastewater surveillance for infectious human and/or animal diseases were included in this study. These infectious diseases could be of viral or bacterial origin. Surveillance was defined as repeated testing over a period of time. Studies triangulating wastewater surveillance data with at least one additional surveillance component, including human clinical cases, animal surveillance, environmental monitoring, or public health response systems, were considered eligible. Formal integration of all three One Health domains (human, animal and environmental health) was not mandatory for inclusion, as this review aimed to evaluate the broader implications of wastewater surveillance for One Health-oriented surveillance systems. Non-peer-reviewed journal articles, like reports or communications of any project working on wastewater surveillance, were also included.

Articles with single-point or one-day wastewater surveillance were excluded from the study. Studies that did not triangulate WWS data with other sources were excluded. Systematic reviews, literature reviews or meta-analyses were excluded.

### 2.3. Selection of the Articles

Articles obtained from different databases were uploaded to Covidence (https://www.covidence.org/) for a seamless screening process. Duplicates were removed by the software itself. The first round of screening titles and abstracts was performed independently by two reviewers, and for the second round, two reviewers selected full-text articles as per the selection criteria. Any conflict was resolved by a third reviewer after discussion.

### 2.4. Assessment of Methodological Quality

We utilized specific Joanna Briggs Institute (JBI) checklists tailored for each study type: cross-sectional studies and any non-peer reviewed articles. Any disagreement that arose between the reviewers was resolved through discussion. The JBI Critical Appraisal checklists for analytical cross-sectional studies and other study design has been attached in the Supplementary File S3.

### 2.5. Data Extraction

Data extraction of the selected full-text articles was conducted by reviewers in predesigned proforma that included author, country, pathogen types, infectious disease, method of identification, prevalence of pathogen, findings, etc.

### 2.6. Data Analysis

The context, study design, setting and type of outcomes of the included individual studies were different and heterogenous, and it was therefore difficult to perform meta-analysis for this systematic review. However, we summarized the context of all selected articles to determine the overall concept related to wastewater surveillance.

## 3. Results

**Figure 1:**
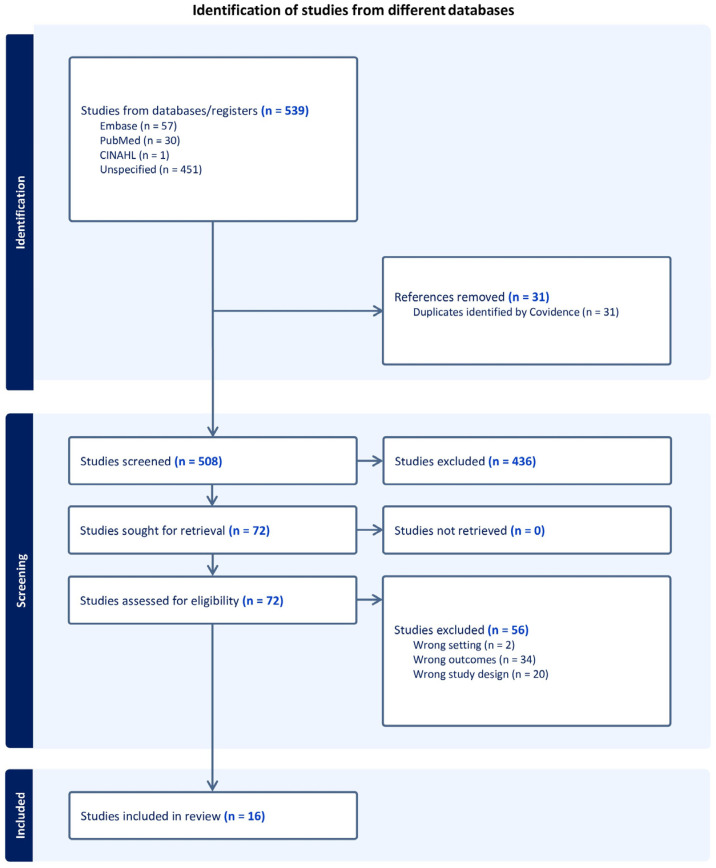
PRISMA flow diagram.

The search was conducted on 26–28 October 2024, and the complete screening process is represented in the Preferred Reporting Items for Systematic Reviews and Meta-Analyses (PRISMA) flow diagram (Figure 1). Initially, 539 studies were retrieved and after deduplication of 31 articles, 508 articles were further screened. After applying title and abstract inclusion criteria, 72 articles met the requirements. After the first round of screening, 56 articles were excluded from pre-defined criteria and only 16 articles were included for the qualitative synthesis (Table 1) (Supplementary File S4).

### 3.1. Study Characteristics

Among the 16 included articles, 11 were conducted in the high-income countries USA (5), Canada (3), Uruguay (1), Israel (1), and Italy (1), and 5 were from low–middle-income countries including India (1), Brazil (1), Bangladesh (1), Tunisia (1), and South Africa (1). Out of 16 studies, only 2 were conducted in the Southeast Asia region.

Data extracted from the included studies revealed an array of pathogens associated with respiratory diseases, gastrointestinal infections and several antimicrobial resistance genes (ARGs). The predominant pathogens identified were from the *Coronaviridae, Caliciviridae* and *Picornaviridae* families. Wastewater surveillance detected the following pathogens: SARS-CoV-2 (10), *Enterovirus* (2), *Hepatitis* E (1), *Influenza* A (1), Pepper mild mottle virus (1) and Urinary enteric virus (1). Most of the studies were conducted during the COVID-19 pandemic period. In addition to SARS-CoV-2, other identified viruses were responsible for acute nonbacterial gastroenteritis. Different PCR techniques were used to identify pathogens in wastewater samples.

In the included studies, the sample collection sites were primarily wastewater treatment plants; however, in four studies, the sample collection sites were defined communities such as universities, hospitals, or neighbourhoods. Viruses identified from these studies were transmitted through food and person-to-person via a fecal–oral route.

### 3.2. Comparison of WWS Cases with Clinical Cases

Many studies compared wastewater surveillance findings with clinical and environmental surveillance indicators, including reported human and animal cases during the same period. The varying prevalence of pathogens across different wastewater matrices illustrated the potential for continuous exposure and transmission within communities. For instance, a study by Bueno et al. in Brazil showed a significant positive correlation (95% confidence level, *p* < 0.05) between viral load in sewage and clinical cases, with Spearman’s rho correlation coefficient ranging from 0.41 to 0.61 [14]. Similarly, Erster et al. in Israel revealed a surge in Enterovirus D68 (EVD68) circulation in wastewater samples, which mirrored clinical cases in the residential area, signalling an early warning for a potential outbreak [15]. In Uruguay, Cancela et al. examined human, swine, and wastewater samples and found a high nucleotide identity (94.7% to 95.4%) between the HEV ORF2 partial sequences from humans and ORF2 region between the HEV-8_uy swine strains along with wastewater sample isolates, indicating a shared transmission route [17]. In Italy, Lombardi et al. found a correlation between high SARS-CoV-2 detection in bivalve mollusks and wastewater samples, suggesting that the presence of SARS-CoV-2 RNA in mollusks could be an indicator of viral circulation in wastewater [20]. Similarly, in the study by Tierney et al., a significant positive correlation (*p* < 0.05) was found between overall antibiotic prescriptions in hospitals and the number of antimicrobial resistance genes (ARGs) detected in hospital wastewater across various community sites, with a Pearson correlation coefficient of 0.25 [26]. Similar findings were observed by Rajput et al., where a strong correlation for the entire city’s viral load exhibits a significant association with daily clinically reported COVID-19 positive cases (R2 = 0.81, *p* < 22 × 10^−16^) [23]. These studies underscore the role of wastewater surveillance in tracking pathogens and early warning for disease outbreaks.

### 3.3. Seasonal Trend of Pathogen Circulation

The included studies identified the following viruses: *Adenovirus*, *Norovirus*, *Enterovirus*, *Hepatitis* A and E, and *Astrovirus*. These viruses have a fecal–oral route of transmission responsible for causing outbreaks with seasonal trends. Studies have observed seasonal patterns in pathogen circulation, with notable increases during specific months. A study by Hubert et al. in Alberta, Canada, demonstrated that the emergence of the Omicron variant drove a surge in clinical cases during December and January [18]. The results mirrored the shift from the Delta to the Omicron variant, with Omicron levels exceeding 50% by 16 December and over 95% by 28 December. Similarly, a Smith et al. study identified many viral circulations like human *Adenovirus*, *Influenza* A virus, human *Rhinovirus*, human *Parainfluenzavirus*, respiratory syncytial virus, human *Parechovirus* and human *Metapneumovirus* following a cyclical pattern [25]. Samples collected from wastewater over a shorter duration showed the most dissimilar patterns—for example, spring vs. fall or winter vs. summer. In contrast, samples collected over a longer duration, cycling back to the same season, exhibited more similarity. These findings indicate that the viral community in winter 2021 closely resembled that of winter 2020. In South Africa, Ngqwala et al. found that viral loads peaked during the winter months, with the Omicron variant becoming dominant from late spring to mid-summer, resulting in a significant rise in infections [21].

### 3.4. Relationship Between Social Vulnerability and Wastewater Surveillance (WWS)

Studies have highlighted a correlation between socioeconomic factors and wastewater surveillance (WWS) results. A study by Akingbola et al. on shelter homes and transient facilities demonstrated that frequent wastewater sampling provided early warning signs of infections, often preceding clinical case detection [13]. Similarly, Bueno et al. found correlations between viral load and different socioeconomic contexts, particularly in more populous areas [14]. Ngqwala et al. observed fluctuations in viral load in low-income or peri-urban communities with insufficient sanitation infrastructure, where occasional releases of inadequately treated wastewater might contribute to increased viral presence in the environment [21].

### 3.5. Implications for Integrated Surveillance

Wastewater surveillance provided valuable insights about community health, where enhanced coordination between state, central and intersectoral agencies strengthen early warning outbreaks and responses. An example is a study conducted by Bueno et al. in Brazilian cities at the beginning of the COVID-19 pandemic, where regular monitoring of wastewater allowed the identification of hotspots and provided data for the early warning of new outbreaks [EWS] through enhanced clinical testing, tracking asymptomatic and oligosymptomatic individuals, and tailoring additional preventive strategies [14]. All data were regularly shared with the Ministry of Health and local health secretariats to complement epidemiological surveillance and strengthen the COVID-19 pandemic response. The weekly report published by the government for public consultation supported a prompt public response. Similarly, a study by Akingbola et al. highlighted the importance of collaboration between facility managers and public health authorities in using wastewater surveillance for early outbreak detection [13]. The first positive signal prompted a joint assessment and proactive response, with continuous communication leading to targeted testing and control measures.

## 4. Discussion

This systematic review has synthesized evidence from 16 studies covering diverse geographical, epidemiological and infrastructural contexts confirming WWS as a population level sentinel tool for infectious disease surveillance. The aggregated findings pose a particular question regarding action thresholds, analytical sensitivity, scalability and equitable implementation across varying resource environments. There is enough evidence to support the correlation between WWS and community disease burden in high-income countries, but the translation of these associations into actionable public health decisions for low- and middle-income countries requires a more structured interpretive framework. These findings highlight the potential utility of wastewater surveillance in supporting early outbreak detection and public health preparedness. It also critically examines the interpretive, methodological and systemic dimensions of WWS, drawing on cross study comparisons to identify both the promise and the unresolved challenges of this evolving surveillance modality.

About two-thirds of the included articles in the study have focused on SARS-CoV-2, which highlights the global recognition of wastewater surveillance as a non-invasive and community-level indicator of disease prevalence. This is both a strength and a limitation of the current evidence base. While the COVID-19 pandemic accelerated methodological refinement and validated the epidemiological sensitivity of WWS at an unprecedented scale, this concentration risks overfitting the surveillance paradigm to a single and well-characterized pathogen with high fecal shedding rates and established molecular targets. The sensitivity of RT-qPCR-based SARS-CoV-2 detection in wastewater is heavily dependent on shedding dynamics that vary considerably across infection phases, viral variants and individual host factors [29]. It provides a confounding dimension that is rarely controlled for in wastewater studies. This observation is also corroborated by findings from Holland et al., who demonstrated the utility of adenovirus detection in wastewater and its correlation with clinical presentation on a university campus [30]. Hubert et al. observed that the emergence and predominance of the Omicron variant were mirrored in wastewater samples, providing an early indication of shifts in variant dominance [18]. Similarly, Rios et al.’s application of whole-genome sequencing for lineage abundance estimation illustrates the potential of genomic WWS, but also underscores a scalability gap [31]. Technically, such approaches require bioinformatic infrastructure and technical expertise that remain concentrated in academic research centres rather than operational public health agencies. Moreover, studies detecting enterovirus, hepatitis E virus and antimicrobial resistance genes (ARGs) further exemplify the versatility of WWS [32,33,34]. Another finding of this study is consistent with Piggot et al.’s work focusing on WWS’s capacity to tactically identify WW signals correlated with clinical cases in high-risk senior congregate living homes, which notify the importance of public health measures and risk communication in this kind of setting [35].

The cross-study pattern of elevated pathogen signals during winter months, which was documented consistently by Smith et al., Ngqwala et al., Tiwari et al., and Wang et al. validates the expected seasonal epidemiology of enteric and respiratory pathogens [21,25,36,37]. This also exposes a methodological confound that is systematically underexplored in this literature, such as the effect of temperature, precipitation and flow rate on wastewater matrix composition and viral stability. Cold temperatures retard viral RNA degradation, thereby artificially elevating measured concentrations in winter relative to actual shedding, while heavy rainfall events cause hydraulic dilution that can suppress signal detection below analytical thresholds [38,39]. Both phenomena are capable of generating spurious seasonal trends if not accounted for through flow-normalization. The observation by Wang et al. that norovirus GII was disproportionately prevalent in wastewater relative to other enteric pathogens was particularly instructive, as this likely reflects the extraordinarily high fecal shedding titres of *Norovirus* (up to 10^13^ genome copies per gram of stool) rather than a true ecological predominance, illustrating the hazard of interpreting raw wastewater concentration data as a proxy for disease incidence without pathogen specific shedding correction factors [37,40,41,42]. The implications for actionable public health decision-making are significant if seasonal signal fluctuations cannot be reliably attributed to changes in community transmission as opposed to environmental matrix effects, and the utility of WWS as a trigger for time-sensitive interventions is substantially diminished. Future methodological consensus efforts should consequently prioritize standardized flow-normalized reporting measures that are comparable to air quality index systems.

The geographic diversity of the included studies ranged from high-income countries (USA, Canada, Italy, Israel) to low- and middle-income countries (India, Brazil, South Africa, Bangladesh), which demonstrated the universal applicability of WWS. However, there are lot of disparities in its implementation strategies. WWS is implemented within centralized, piped sewerage networks where catchment boundaries are precisely defined, flow rates are metered and sample representativeness can be statistically modelled. This architectural prerequisite underpins the quantitative accuracy upon which epidemiological inference depends. In contrast, the predominant sanitation topology across LMICs is characterized by fragmented networks, open drainage channels, pit latrines and septic systems with variable and largely unmeasured discharge pathways, which renders core assumptions of centralized WWS methodologically challenging [43,44,45]. Furthermore, the strong wastewater-clinical correlations reported in studies from high-income countries [15,17,46,47] should not be generalized to LMIC contexts, as the clinical surveillance systems in these settings are themselves characterized by low testing coverage and significant underreporting. Addressing this problem requires the development of context-adapted WWS frameworks, which includes neighbourhood-level grab sampling from open drains, the use of environmental fecal indicator bacteria as denominators, and community calibrated shedding models rather than the mere use of protocols from high-income countries into resource limited environments.

Despite conducting this review within a One Health perspective, only a limited number of included studies explicitly operationalized integrated human, animal, and environmental surveillance systems. Most studies focused primarily on wastewater monitoring linked to human clinical surveillance, while the incorporation of animal health surveillance and formal intersectoral governance mechanisms remained limited. This is not merely a theoretical deficiency. It has direct consequences for the interpretive completeness of wastewater signals, particularly in the context of zoonotic pathogens and antimicrobial resistance. For ARGs, wastewater signals integrating human and livestock antibiotic usage patterns cannot be reliably disaggregated into source-specific contributions without concurrent veterinary and environmental sampling, yet none of the included studies attempted such decomposition [34]. The Brazil case study illustrates both the promise and the ceiling of current practice, while geographic hotspot mapping from SARS-CoV-2 wastewater data effectively guided targeted community testing, and the response framework operated solely within the human health vertical with no documented feedback loop to environmental or animal health agencies [14]. This approach facilitated targeted testing and supported the development of preventive measures tailored to specific communities [48]. Similarly, a study in Israel demonstrated the detection of *Enterovirus* D68 (EVD68) in wastewater-paralleled clinical cases, highlighting its role as a sentinel surveillance system [15]. This action threshold gap is the most consequential operational deficit in the current WWS literature. In the absence of clear evidence-based criteria for escalation, WWS data might function as epidemiological intelligence rather than a decision support system. Future initiatives must incorporate pre-established and tiered response triggers within a formalized One Health governance structure that incorporates intersectoral data sharing protocols across human, veterinary and environmental surveillance pillars. These triggers should be based on pathogen-specific shedding models, local clinical testing rates and healthcare capacity indices.

The methodological heterogeneity documented across the included studies represents a more fundamental challenge than is typically acknowledged. It is not simply a matter of protocol inconvenience but a direct threat to the inter-study comparability upon which surveillance networks depend. Sensitivity and specificity limitations in WWS operate at multiple and compounding levels. At the pre-analytical stage, sample concentration methods that include ultrafiltration, polyethylene glycol precipitation and electronegative membrane filtration differ substantially in recovery efficiency across pathogens and matrix conditions, with reported recoveries ranging from below 10% to over 90% for the same target, depending on the method and wastewater composition [49]. At the analytical stage, the choice of RT-qPCR targets, primer-probe sets and amplification parameters introduces inter-laboratory variability that can produce concentration estimates differing by one to two orders of magnitude for identical samples [50]. The absence of certified reference materials and external quality assurance schemes analogous to those used in clinical diagnostics means that no current WWS dataset can be considered metrologically traceable, thereby severely limiting its utility for cross-jurisdictional or longitudinal comparison. Specificity is an equally underexamined concern, as environmental inhibition, cross-reactive amplification of closely related nucleic acid sequences, and the detection of non-infective RNA from inactivated viral particles can all generate false-positive signals [51]. This is a particularly consequential error type when wastewater surveillance is used to trigger costly public health interventions. The discrepancies between WWS data and clinical case counts observed across several studies should therefore be interpreted not simply as a communication gap but as a signal that the quantitative relationship between wastewater viral load and population-level infection prevalence remains complex and highly content-dependent [14,52]. The epidemiological conversion factor central to WWS inference is poorly characterized and context-dependent [53]. Establishing this relationship rigorously, through prospective studies that combine individual-level shedding data with catchment-level wastewater measurements and clinical case ascertainment, must be recognized as a foundational research priority before WWS can be confidently operationalized as a decision-support tool.

The capacity of WWS to detect asymptomatic and oligosymptomatic infections is an epidemiological advantage made particularly salient during the COVID-19 pandemic [13,14]. WWS provides marginal additional case-detection benefit, whereas in settings with low testing coverage like LMICs, WWS could theoretically capture a substantial proportion of community transmission that is entirely invisible to clinical systems [54]. However, this potential is undermined by the same sewer infrastructure deficits that limit representativeness in these settings, creating a paradox wherein the populations with the greatest need for supplementary surveillance are least amenable to the current WWS model. The claim that WWS is inherently cost-effective and scalable also deserves analytical scrutiny [55]. Scalability is contingent on the availability of centralized laboratory infrastructure, cold-chain logistics for sample transport and bioinformatics capacity for data interpretation—none of which are available in resource limited environments [56]. The integration of WWS with genomic epidemiology to track variant emergence and zoonotic events is an area of genuine and expanding utility, but current practice suffers from a critical integration deficit [17,20,57]. Wastewater genomic data and clinical surveillance data are typically generated, managed and interpreted by different institutions with incompatible data architectures, and no included study described a formalized, prospective data-sharing protocol governing this integration. Without such structures, real-time genomic intelligence from wastewater is systematically delayed in reaching clinical decision-makers, negating much of the early warning advantage that the technology affords [58].

Emerging technological advances including AI-assisted signal interpretation [27,59,60] and next-generation sequencing (NGS) for untargeted pathogen characterization [16,61] hold genuine transformative potential for WWS, but their deployment raises critical questions about the sequencing of innovation and infrastructure that the literature has not adequately engaged. AI and machine learning models trained on wastewater time-series data from high-income countries and centralized sewerage systems cannot be validly transferred to fragmented or informal drainage networks without substantial retraining and validation. Consequently, the logistical and financial barriers to generating such training datasets in LMICs are formidable [62]. Similarly, while NGS enables hypothesis-free pathogen discovery and detection of variants of concern in wastewater, its computational demands, long turnaround times relative to RT-qPCR, and sensitivity limitations at low viral concentrations currently preclude its use as a routine operational surveillance tool. It remains most appropriately positioned as a second-tier confirmatory or discovery instrument. The argument that WWS investment constitutes a cost-effective pandemic prevention strategy is compelling in aggregate economic modelling [19,22,24,28,63], but it masks very important distributional questions. For instance, countries that were least able to absorb pandemic economic shocks are those most likely to lack the sewage infrastructure, laboratory capacity, and governance systems needed to implement effective WWS. Without deliberate multilateral financing mechanisms and technology transfer provisions, advancement in WWS risks widening rather than closing the global health security divide. Critically, future methodological development must prioritize the establishment of standardized, internationally benchmarked protocols for covering sample collection, concentration, pathogen quantification, data normalization and reporting metrics as a precondition for building the interoperable global WWS networks that pandemic preparedness demands.

Capacity building in LMICs must be reconceptualized as a structural rather than supplementary priority. Effective WWS in low-resource settings requires not only laboratory personnel training but the concurrent development of sampling infrastructure adapted to non-centralized sanitation systems, locally validated analytical protocols, context-specific epidemiological conversion models and functional data governance frameworks that connect wastewater intelligence to national public health decision chains. International organizations such as the World Health Organization (WHO) and United Nations Environment Programme (UNEP) and bilateral donors must move beyond short-term technical assistance toward sustained investment in nationally owned WWS institutions with standardized procedures for competency development and quality-assurance systems that enable LMICs to participate meaningfully in global surveillance networks rather than functioning as passive data contributors to research conducted primarily by high-income academic partners. Ultimately, the long-term credibility and policy relevance of WWS as a global health security instrument will depend not on further proof-of-concept demonstrations in well-resourced settings, but on demonstrating that its core analytical and operational assumptions are valid, transferable and actionable across the full spectrum of epidemiological, infrastructural and governance environments in which the next pandemic is most likely to emerge.

## 5. Conclusions

Wastewater surveillance (WWS) is an important complementary approach to monitor pathogen circulation and can help in understanding population-level infectious disease trends. The evidence synthesized in this review suggests that WWS can support the early detection of circulating pathogens and provide useful epidemiological insights when it is integrated with clinical and environmental surveillance systems. However, the ability of WWS to consistently predict emerging variants or directly prevent outbreaks remains context-dependent and influenced by methodological, infrastructural, and surveillance-related factors. The findings highlight the relevance of WWS within a One Health framework, particularly for community-level monitoring and outbreak preparedness. Further standardization of methodologies, stronger integration with public health systems, and expanded implementation in diverse settings are required to strengthen its utility for public health decision-making and preventive action.

## Figures and Tables

**Table 1:** Main characteristics of the studies included in the systematic review.

Study	Year	Total Samples	Methods	Detected Pathogen	Domain	Clinical Correlation
Akingbola et al. [13]	2021	Wastewater from shelter with 169 residents and 105 staff	RT-qPCR	SARS-CoV-2	Wastewater surveillance alerted to the presence of COVID-19 activity at the site, prior to clinical detection.	No
Bueno et al. [14]	2021–2022	-	RT-qPCR	SARS-CoV-2	The peaks of new cases in the second and third waves of COVID-19 coincided with the peaks of viral RNA concentration in the wastewater samples.	Yes
Erster et al. [15]	2021	29 wastewater samples and 472 hospitalized pediatric patients	RT-qPCR	Enterovirus D68	Wastewater-based surveillance may be used as a complementary tool for continuous monitoring of *Enterovirus* D68 circulation, in parallel with testing of suspicious clinical cases.	Yes
Haque et al. [16]	2021	504 wastewater samples	RT-qPCR and Genome sequencing	SARS-CoV-2	Early detection through wastewater monitoring can give public health officials supplementary information to rapidly triangulate the prevalence of circulating variants within a community.	Yes
Cancela et al. [17]	2020–2023	92 wastewater samples	RT-nested PCR and Next Generation Sequencing (NGS)	Hepatitis E Virus	The study was unique in terms of the molecular epidemiology and transmission paths of HEV, showing how a single circulating subtype has been identified in humans, animal reservoirs and hosts, and environmental samples.	No
Hubert et al. [18]	2021–2022	233 wastewater samples	RT-qPCR	SARS-CoV-2 Omicron variant	Wastewater surveillance results demonstrated that the emergence of Omicron was the driver of clinical cases increasing in December 2021 and January 2022.	Yes
Li et al. [19]	2022	48 wastewater samples	PCR and NGS	*Orthopoxvirus*, *Rhadinovirus*, *Parapoxvirus*, *Varicellovirus*, *Hepatovirus*, *Simplexvirus*, *Bocaparvovirus*, *Molluscipoxvirus*, *Parechovirus*, *Roseolovirus*, *Lymphocryptovirus*, *Alphavirus*, *Spumavirus*, *Lentivirus*, *Deltaretrovirus*, *Enterovirus, Kobuvirus*, *Gammaretrovirus*, *Cardiovirus*, *Erythroparvovirus*, *Salivirus*, *Rubivirus*, *Orthohepevirus*, *Cytomegalovirus*, *Norovirus* and *Mamastrovirus*. *Astrovirus*, *Betapolyomavirus*, *Norovirus*, and *Enterovirus*	-	Yes
Lombardi et al. [20]	2021–2022	168 wastewater samples and 57 mollusk samples	RT-qPCR	SARS-CoV-2	The results of this study confirm that environmental surveillance has the potential to document the diffusion of the virus and suggest the use of these samples for monitoring purposes.	No
Ngqwala et al. [21]	2022–2023	-	RT-qPCR	SARS-CoV-2	Frequent monitoring of the wastewater-surveillance-based studies provide early warning signals for the proliferation of SARS-CoV-2.	Yes
Othman et al. [22]	2021–2022	44 wastewater samples	Solid digital PCR and whole-genome sequencing	SARS-CoV-2	The study reports the first detection of Delta and Omicron variants in wastewater in Tunisia in WWTP influent samples before the increment of clinically diagnosed new COVID-19 cases.	Yes
Rajput et al. [23]	2021–2022	442 wastewater samples	RT-qPCR	SARS-CoV-2	The study highlighted the efficacy of wastewater surveillance (WWS) as a formidable tool in the early warning, detection, and monitoring of SARS-CoV-2 variants.	Yes
Scott et al. [24]	2022–2023	-	qRT-PCR	*Influenza* A	The study demonstrates how wastewater surveillance can shed light on regional differences that may have otherwise gone unnoticed, or remain unvalidated, because of the inherent limitations of traditional metrics to capture population-wide trends.	
Smith et al. [25]	-	1408 wastewater samples	-	*Polyomaviridae*, *Picornaviridae*, *Astroviridae*, *Caliciviridae*, and *Coronaviridae* SARS-CoV-2, *Influenza* A	-	Yes
Tierney et al. [26]	2020–2022	2238 wastewater samples	RT-qPCR and RNA sequencing	SARS-CoV-2 and enteric pathogens	-	Yes
Tisza et al. [27]	-	363 wastewater samples	sequencing-based	SARS CoV- 2, Influenza virus, and Monkeypox viruses	-	Yes
Yaglom et al. [28]	2021	-	Genome sequencing	SARS-CoV-2	Understanding transmission patterns within the outbreak population helps identify improved disease prevention and mitigation strategies, increasing community resilience for future outbreaks.	Yes

## Data Availability

No new data were created or analyzed in this study. All data supporting the findings of this review are available within the article and its Supplementary Materials.

## Supplementary Materials

The following supporting information can be downloaded at: https://www.mdpi.com/article/10.3390/pathogens15070690/s1.

## Abbreviations

The following abbreviations are used in this manuscript:

WWS	Wastewater surveillance
AMR	Antimicrobial resistance
PRISMA	Preferred Reporting Items for Systematic Reviews and Meta-Analyses
JBI	Joanna Briggs Institute
