# Supplementary material for: Wastewater Surveillance for Early Warning of Infectious Disease Outbreaks: A Systematic Review of Evidence and Implications for One Health Surveillance
Source: Pathogens. 2026 Jun 30;15(7):690. doi: 10.3390/pathogens15070690 (PMC13416211; doi:10.3390/pathogens15070690)
Supplement: Supplementary file 1 [file pathogens-15-00690-s001.zip › Supplementary File S2.pdf]

Effectiveness of Wastewater Surveillance under One-Health Framework in Prevention and Early Warning of Disease Outbreaks- A systematic Review

Concept	Search terms	Results
#1 waste water surveillance	(((((((((("Wastewater-Based Epidemiological Monitoring"[MeSH Terms]) OR ("surveillance wastewater based"[Title/Abstract])) OR ("wastewater based surveillance"[Title/Abstract])) OR ("sewage based epidemiology"[Title/Abstract])) OR ("surveillance sewage"[Title/Abstract])) OR ("Sewage-Based Surveillance"[Title/Abstract])) OR ("wastewater epidemiology"[Title/Abstract])) OR ("Wastewater Based Epidemiology"[Title/Abstract])) OR ("Surveillance, Wastewater"[Title/Abstract])) OR ("Environmental Monitoring"[MeSH Terms])) OR ("Environmental Surveillance"[Title/Abstract])) OR ("event based surveillance"[Title/Abstract])	184,145
#2 Disease outbreak	((((((((((((((((((("Disease Outbreaks"[Mesh]) OR ("Pandemics"[Mesh])) OR ("Epidemics"[Mesh])) OR ("Disease Transmission, Infectious"[Mesh])) OR ("Infectious Disease Transmission, Patient-to-Professional"[Mesh])) OR ("Disease Outbreak"[Title/Abstract])) OR ("Outbreak, Disease"[Title/Abstract])) OR ("Outbreaks, Disease"[Title/Abstract])) OR ("Infectious Disease Outbreaks"[Title/Abstract])) OR ("Disease Outbreaks, Infectious"[Title/Abstract])) OR ("Infectious Disease Outbreak"[Title/Abstract])) OR ("Outbreak, Infectious Disease"[Title/Abstract])) OR ("Outbreaks, Infectious Disease"[Title/Abstract])) OR ("Epidemics"[Title/Abstract])) OR ("Epidemic"[Title/Abstract])) OR ("Pandemics"[Title/Abstract])) OR ("Pandemic"[Title/Abstract])) OR ("Disease surveillance"[Title/Abstract])) OR ("Early prediction"[Title/Abstract])) OR ("outbreak prediction"[Title/Abstract])) OR ("Disease monitoring"[Title/Abstract])	565,538
#3 one health	(((((One Health Concept[Title/Abstract])) OR (("One Health"[Mesh]))) OR (("One Health*"[Title/Abstract]))) OR ((One Medicine Initiative[Title/Abstract]))) OR ((One Health Initiative[Title/Abstract]))	14,081
#4<br>#1 AND #2 AND #3	**PubMed**	30

Embase: 57 (Searched on : 26.10.2024)

('Wastewater-Based Epidemiological Monitoring'/exp OR 'surveillance wastewater based':ti,ab OR 'wastewater based surveillance':ti,ab OR 'sewage based epidemiology':ti,ab OR 'surveillance sewage':ti,ab OR 'Sewage-Based Surveillance':ti,ab OR 'wastewater epidemiology':ti,ab OR 'Wastewater Based Epidemiology':ti,ab OR 'surveillance wastewater':ti,ab OR 'Environmental Monitoring'/exp OR 'Environmental Surveillance':ti,ab OR 'event based surveillance':ti,ab) AND ('Disease Outbreaks'/exp OR Pandemics/exp OR Epidemics/exp OR 'disease transmission, infectious'/exp OR 'infectious disease transmission, patient to professional'/exp OR 'Disease Outbreak':ti,ab OR 'outbreak disease':ti,ab OR 'outbreaks disease':ti,ab OR 'Infectious Disease Outbreaks':ti,ab OR 'disease outbreaks infectious':ti,ab OR 'Infectious Disease Outbreak':ti,ab OR 'outbreak infectious disease':ti,ab OR 'outbreaks infectious disease':ti,ab OR Epidemics:ti,ab OR Epidemic:ti,ab OR Pandemics:ti,ab OR Pandemic:ti,ab OR 'Disease surveillance':ti,ab OR 'Early prediction':ti,ab OR 'outbreak prediction':ti,ab OR 'Disease monitoring':ti,ab) AND ('one health concept':ti,ab OR 'One Health'/exp OR 'one health*':ti,ab OR 'one medicine initiative':ti,ab OR 'one health initiative':ti,ab)

Cinhal: 1

((MH "Wastewater-Based Epidemiological Monitoring+") OR (TI "surveillance wastewater based" OR AB "surveillance wastewater based") OR (TI "wastewater based surveillance" OR AB "wastewater based surveillance") OR (TI "sewage based epidemiology" OR AB "sewage based epidemiology") OR (TI "surveillance sewage" OR AB "surveillance sewage") OR (TI "Sewage-Based Surveillance" OR AB "Sewage-Based Surveillance") OR (TI "wastewater epidemiology" OR AB "wastewater epidemiology") OR (TI "Wastewater Based Epidemiology" OR AB "Wastewater Based Epidemiology") OR (TI "surveillance wastewater" OR AB "surveillance wastewater") OR (MH "Environmental Monitoring+") OR (TI "Environmental Surveillance" OR AB "Environmental Surveillance") OR (TI "event based surveillance" OR AB "event based surveillance")) AND ((MH "Disease Outbreaks+") OR (MH Pandemics+) OR (MH Epidemics+) OR (MH "disease transmission, infectious+") OR (MH "infectious disease transmission, patient to professional+") OR (TI "Disease Outbreak" OR AB "Disease Outbreak") OR (TI "outbreak disease" OR AB "outbreak disease") OR (TI "outbreaks disease" OR AB "outbreaks disease") OR (TI "Infectious Disease Outbreaks" OR AB "Infectious Disease Outbreaks") OR (TI "disease outbreaks infectious" OR AB "disease outbreaks infectious") OR (TI "Infectious Disease Outbreak" OR AB "Infectious Disease Outbreak") OR (TI "outbreak infectious disease" OR AB "outbreak infectious disease") OR (TI "outbreaks infectious disease" OR AB "outbreaks infectious disease") OR (TI Epidemics OR AB Epidemics) OR (TI Epidemic OR AB Epidemic) OR (TI Pandemics OR AB Pandemics) OR (TI Pandemic OR AB Pandemic) OR (TI "Disease surveillance" OR AB "Disease

surveillance") OR (TI "Early prediction" OR AB "Early prediction") OR (TI "outbreak prediction" OR AB "outbreak prediction") OR (TI "Disease monitoring" OR AB "Disease monitoring")) AND ((TI "one health concept" OR AB "one health concept") OR (MH "One Health+") OR (TI "one health*" OR AB "one health*") OR (TI "one medicine initiative" OR AB "one medicine initiative") OR (TI "one health initiative" OR AB "one health initiative"))

ProQuest: 451

(("Wastewater-Based Epidemiological Monitoring" OR "surveillance wastewater based" OR "wastewater based surveillance" OR "sewage based epidemiology" OR "surveillance sewage" OR "Sewage-Based Surveillance" OR "wastewater epidemiology" OR "Wastewater Based Epidemiology" OR "surveillance wastewater" OR "Environmental Monitoring" OR "Environmental Surveillance" OR "event based surveillance") AND ("Disease Outbreaks" OR "Pandemics" OR "Epidemics" OR "disease transmission, infectious" OR "infectious disease transmission, patient to professional" OR "Disease Outbreak" OR "outbreak disease" OR "outbreaks disease" OR "Infectious Disease Outbreaks" OR "disease outbreaks infectious" OR "Infectious Disease Outbreak" OR "outbreak infectious disease" OR "outbreaks infectious disease" OR "Epidemics" OR "Epidemic" OR "Pandemics" OR "Pandemic" OR "Disease surveillance" OR "Early prediction" OR "outbreak prediction" OR "Disease monitoring") AND ("one health concept" OR "One Health" OR "one health*" OR "one medicine initiative" OR "one health initiative"))
